# Massive Hemoptysis in Children

**DOI:** 10.1155/2020/6414719

**Published:** 2020-06-06

**Authors:** Juan Yang, Fengqin Liu, Yan Liang, Chunyan Guo, Jinrong Wang, Xing Chen

**Affiliations:** ^1^Department of Pediatric, Shandong Provincial Hospital, Cheeloo College of Medicine, Shandong University, Jinan, Shandong 250021, China; ^2^Department of Pediatric, Shandong Provincial Hospital Affiliated to Shandong First Medical University, Jinan, Shandong, 250021, China

## Abstract

*Rationale*. Hemoptysis is a rare but often life-threatening condition in pediatric patients. Massive hemoptysis can easily lead to asphyxia, respiratory failure, shock, and even death. The most common causes of severe hemoptysis are lower respiratory tract infection, vascular malformation, and bronchial foreign body. We present an unusual case of massive hemoptysis caused by malformation of the bronchial artery, which includes bronchial artery hypertrophy, bronchial-pulmonary artery fistula, and ectopic bronchial artery. *Patient*. An 11-year-old boy was admitted to the hospital with mild hemoptysis lasting for the two preceding days. He did not report any discomfort, such as fever or chest pain. His complete blood count and coagulation function were normal. Chest X-ray documented lower right pneumonia. Massive hemoptysis occurred on the night of the admission. *Diagnosis*. Bronchial arteriography revealed that the right lower bronchial artery and the ectopic bronchial artery from the renal artery were the responsible vessels for hemoptysis. *Interventions*. The boy underwent a successful bronchial artery embolization and bronchoscopy to remove the blood clot from the airway. *Outcomes*. After bronchial artery embolization and bronchoscopy, the boy recovered without complications. Hemoptysis and chest pain disappeared, and chest radiographs returned to normal. *Lessons*. Bronchial arterial bleeding often presents as life-threatening massive hemoptysis. Patients should immediately receive hemostatic treatment and undergo chest CTA, bronchial arteriography, BAE, and bronchoscopy according to their condition. Rapid identification of the etiology and symptomatic treatment are critical to saving the lives of children.

## 1. Introduction

Vascular malformations of the pulmonary and bronchial arteries are one of the most important causes of hemoptysis. Common types of malformations include pulmonary atresia, pulmonary arteriovenous fistula, bronchial-pulmonary artery fistula, and aortopulmonary collateral vessels. Bronchial arteries are involved in 90% of incidences of severe hemoptysis and vary in terms of origin, number, and anatomic course. The bronchial artery usually originates from the descending aorta at the level of the t5-t6 vertebral body. An artery arising at another level is considered an ectopic bronchial artery [1]. Ectopic bronchial artery dilatation from the renal artery accompanied by bronchial-pulmonary artery fistula has not been reported thus far. Here, we report the clinical presentation, treatment, and prognosis in a child with massive hemorrhage due to bronchial artery malformation.

## 2. Clinical Experience

**Figure 1:**
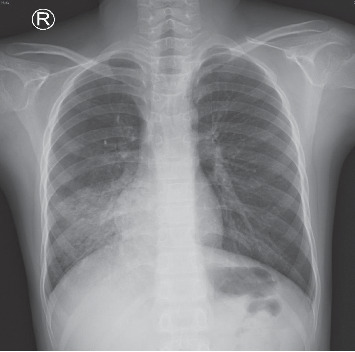
Chest X-ray documented right lower pneumonia.

An 11-year-old boy was admitted to the pediatric respiratory department of the Shandong Provincial Hospital due to 2-day history of intermittent mild hemoptysis. Six days earlier, the child had a fever with the highest temperature of 38.0°C. He had no other symptoms, and no examination was performed or treatment was given. Three days before the admission, the child was accidentally injured in the abdomen by the elbow of a classmate while playing. The resulting pain subsided soon, and no special treatment was given. The next day, the boy began coughing up bright red or dark red blood, 2–4 times a day, with a 30–40 mL of blood loss over 24 hours. He did not experience heart palpitations, nosebleed, skin bleeding, coma, hematuria, bloody stool, weight loss, or other symptoms. The boy had been full-term at birth, healthy, and without a history of tuberculosis or measles. At the time of admission, the patient was not febrile and had a respiratory rate of 30 breaths/min, heart rate of 92 beats/min, blood pressure of 107/80 mmHg, and oxygen saturation of 99% in ambient air. The weight was 35 kg. The breath sound of the right lung was slightly lower, and the results of other physical examinations were unremarkable. The complete blood count showed white blood cells 10.18 × 10^9^/L, hemoglobin 141 g/L, and platelets 324 × 10^9^/L, and the C-reactive protein (CRP) level was less than 0.2 mg/L. The coagulation function was normal. The chest radiograph documented right lower pneumonia (Figure 1).

**Figure 2:**
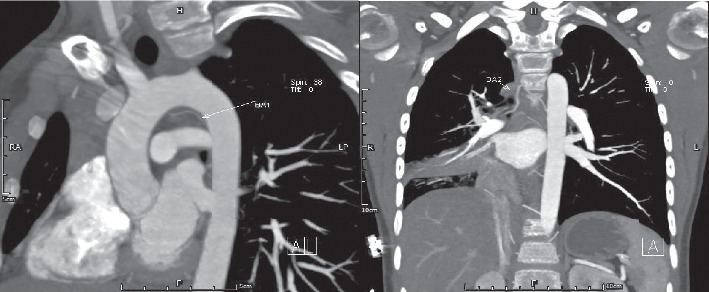
CTA demonstrated that BA1 originated from the thoracic aorta to the left at the level of the upper margin of T5 and entered the right hilum above the bifurcation of the trachea. BA2 arose from the right side of the thoracic aorta at T6 and passed up to the right and then down to the right hilum, with a widening of the diameter (2 mm) of the primary and middle tubes.

After the admission, the boy was treated by ceftriaxone, vitamin K1, and etamsylate and was told to remain in bed and avoid strenuous activity. On the night of admission, hemoptysis occurred more than 10 times, with 20–30 ml of blood each time. The events were accompanied by obvious chest tightness and pain. Physical examination revealed body temperature 36.8°C, heart rate 120 beats/min, respiratory rate 35 breaths/min, blood pressure 118/92 mmHg, oxygen saturation of 98% in ambient air, and low breathing sounds in the right lung. According to the result of chest radiography and the reduction in the right lung breath sound, the possibility of right lung hemorrhage was considered. The vital signs of the boy were stable, and no signs of respiratory failure were present. Therefore, the patient was subjected to the administration of oxygen via a nasal catheter, ECG monitoring, infusion of hemocoagulase, and continuous administration of pituitrin. He was placed in a right lateral position to protect the airway. Auxiliary examination indicated normal blood count (white blood cells 10.22 × 10^9^/L, hemoglobin 134 g/L, platelets 337 × 10^9^/L). Normal values were obtained in liver function tests, biochemical tests, myocardial enzyme test, urine test, antistreptolysin O test, autoantibodies, immunoglobulin, complement, prothrombin time, activated partial thromboplastin time, and erythrocyte sedimentation rate. The tests for pathogens including bacteria, mycoplasma, tuberculosis, and fungi were all negative. Cardiac ultrasound was normal. Acute CT angiography (CTA) showed that bronchial artery 1 originated from the thoracic aorta to the left at the level of the upper margin of T5 and entered the right hilum above the bifurcation of the trachea. Bronchial artery 2 arose from the right side of the thoracic aorta at T6 and passed up to the right and then down to the right hilum with a 2 mm widening of the primary and middle tube diameters (Figure 2). Bronchial artery 3 originated from the left side of the thoracic aorta under T6 and was divided into two branches going, respectively, to the right and left, without an evident expansion of the lumen. The pulmonary CTA did not identify significant abnormalities. The chest CTA also showed intra-alveolar hemorrhage, partial bronchial stenosis on the right side, atelectasis in the middle lobe, and partial consolidation in the lower lobe. The interventional therapy department physicians concluded that the massive hemoptysis might be caused by bronchial artery dilatation, and bronchial arteriography was recommended to obtain a definite diagnosis, with bronchial artery embolization (BAE) if necessary.

**Figure 3:**
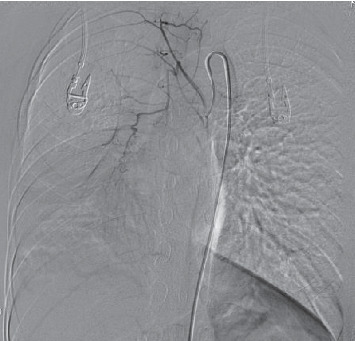
Angiography showed that the right lower bronchial artery had the same origin as the left bronchial artery, and the vascular lumen was dilated. The right lower lung branches were disorganized, with a small amount of light staining in the parenchymal phase.

**Figure 4:**
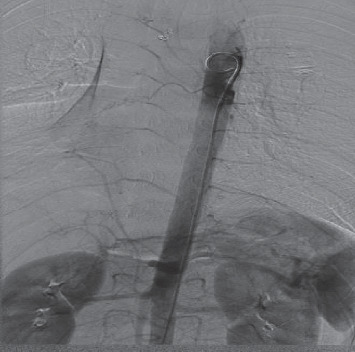
Aortic angiography showed an ectopic bronchial artery originating from the right renal artery and entering the right lower lung with tortuous and disordered peripheral vessels; the pulmonary artery branch is shown in the arterial stage.

**Figure 5:**
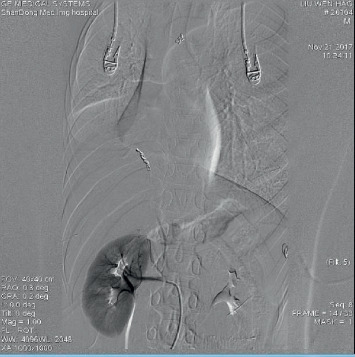
The end of the ectopic bronchial artery is blocked.

Bronchial arteriography was performed 4 hours after the discontinuation of pituitrin. The imaging demonstrated that the right lower bronchial artery had the same origin as the left bronchial artery, and the vascular lumen was dilated. The right lower lung branches were disorganized, with a small amount of light staining in the parenchymal phase (Figure 3). The distal end of the right inferior bronchial artery was embolized by microspheres combined with a metal coil, this occlusion was confirmed by a repeated bronchial arteriography. Aortic angiography visualized an ectopic bronchial artery originating from the right renal artery and entering the right lower lung, with tortuous and disordered peripheral vessels, and the pulmonary artery branch was apparent in the arterial phase (Figure 4). In consideration of the presence of a bronchial-pulmonary artery fistula, embolization was performed at the distal end of the vessel (Figure 5), and the subsequent revealed that the artery was occluded at the distal end. On this basis, it was concluded that bronchial artery 3 and the ectopic bronchial arteries originating from the kidney were the vessels responsible for the bleeding, and not the slightly dilated bronchial artery 2.

**Figure 6:**
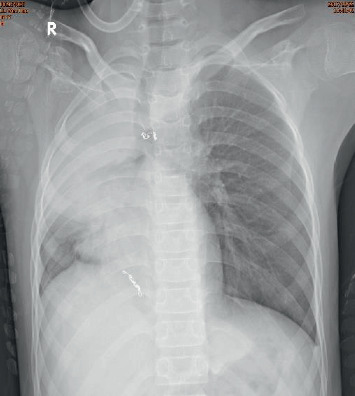
Chest X-ray documented atelectasis in the right lung.

**Figure 7:**
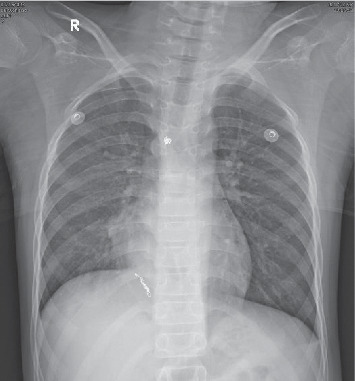
One day after bronchoscopy, no evidence of atelectasis was found on the chest X-ray.

No incidence of hemoptysis occurred after BAE, but the child continued to have paroxysmal chest tightness and chest pain. Subsequent chest X-ray documented atelectasis in the right lung (Figure 6). To avoid rebleeding, bronchoscopy was performed 2 days after the BAE. A large number of blood clots were found in the right main bronchial lumen, the right upper lobe bronchial lumen, and the right middle bronchial lumen. The thrombus was removed using forceps, bronchoscopic brush, and freezing. After the bronchoscopy, the child did not experience chest tightness or other discomforts and occasionally coughed up a small amount of brown blood clot. The chest radiograph did not reveal any evidence of atelectasis on the second day of bronchoscopy (Figure 7). Bronchoscopy performed again 5 days later did not detect hemorrhagic points or bloody sputum suppository. Given the circumstances preceding the admission, the experienced trauma is considered to be the cause of bleeding from the malformed bronchial artery. The child was hospitalized for 10 days. No incidence of hemoptysis occurred during one and a half years of follow-up, and no abnormality was found in the chest radiograph.

## 3. Discussion

Massive hemoptysis is an acute disease with high mortality and is difficult to diagnose and treat. Among cases of fatal hemoptysis, the inciting cause of death is not the hemorrhagic shock, but asphyxiation and heart failure from the inability to oxygenate or ventilate due to hemorrhage flooding the airways [2]. If left untreated, massive hemoptysis is associated with a mortality rate higher than 50% [1]. Therefore, the diagnostic examination of massive hemoptysis should be performed as soon as possible after the patient's condition is stable.

The incidence of hemoptysis in pediatric patients is rare. The typical etiologies include pneumonia, bronchitis, pulmonary tuberculosis, idiopathic pulmonary hemosiderosis (IPH), bronchiectasis, tuberculosis, and mycotic infections, while connective tissue diseases and pulmonary vascular manifestations are rare [3, 4]. When vascular endothelial cells are damaged, the pulmonary circulatory system usually experiences small-volume bleeding, while the damage in the high-pressure bronchial system causes a large amount of bleeding [5]. In the presented case, according to the patient's medical history, physical examination, and imaging examination, hemoptysis was originally thought to be caused by pneumonia. Despite systemic treatment against infection and hemostasis, the symptoms rapidly deteriorated, resulting in a more severe massive hemoptysis. During the rescue process, the presence of a right pulmonary hemorrhage was considered. However, the patient's vital signs were stable, and there was no indication of respiratory failure. Therefore, the inhalation of oxygen was initiated, and the patient was placed in the right lateral position to protect the ventilation function of the lung. Given the possibility of the development of respiratory failure or hypoxemia with the progression of the disease progresses, preparations were made for endotracheal intubation and emergency bronchoscopy to clear the accumulation of blood in the airway. Meanwhile, the results of a routine blood test were continuously checked, and the blood bank was contacted to prepare blood in the event of hemorrhagic shock. The laboratory results were generally normal, and blood transfusion was not necessary. Pituitrin can be used in the treatment of postpartum hemorrhage, pulmonary hemorrhage, esophageal and gastric varices hemorrhage, and diabetes insipidus [6]. It is also the first-choice medicine in the treatment of massive hemoptysis. During the treatment with pituitrin, abdominal pain, hyponatremia, and increased blood pressure may occur. Therefore, it is necessary to closely monitor biochemical indicators and blood pressure and, if necessary, adjust the dosage in a timely manner [6]. Hemoptysis did not reappear after continuous infusion of pituitrin. In addition, further tests showed no significant signs of infection, and blood test excluded autoimmune and hematological diseases. Therefore, the effort to establish the etiology continued during the treatment of symptoms, and the accurate diagnosis was enabled by CTA and bronchoscopy. To avoid further bleeding during the removal of the blood clot, CTA was performed first to find abnormal vessels and was followed by bronchoarteriography and BAE. Subsequently, bronchoscopy was performed to remove the blood clot from the airway. It should be noted that an ectopic bronchial artery originating from the renal artery, with dilatation and bronchial-pulmonary artery fistula, was found by aortic angiography but not by CTA.

Bronchial arteries are high-pressure systems that supply nutrients to the bronchial walls, which are rich in contractile smooth muscle fibers that respond to physical therapy, such as cold, and to pharmacological treatment [7]. They are connected to the pulmonary arteries through several microvascular anastomoses at the level of the alveoli and respiratory bronchioles [8]. Under the pathological conditions of systemic hypoxemia, alveolar hypoxia, pulmonary infarction, and pulmonary inflammation, a larger volume of oxygenated blood is directed to the ischemic lung through compensatory dilatation of bronchial arteries, the formation of new collateral circulation, and the dilatation of anastomotic vessels [8]. These pathological changes can account for an increased risk of bleeding [8]. Bronchial artery malformation is difficult to diagnose instantaneously due to the absence of specific clinical manifestations. Sudden moderate or severe hemoptysis may be regarded as the first symptom. Chest X-ray or CT may show no clear abnormalities or indicate only the manifestations of pulmonary atelectasis after the block of the respiratory tract by a blood clot, a condition not distinguishable from pneumonia. Moreover, the origin, number, diameter, and route of bronchial arteries vary among individuals, and in some cases identification of the bleeding vessels is difficult. Chest CTA can display images of bronchial arteries and pulmonary arteries, find abnormal bronchial arteries and identify bleeding sites, guide bronchial arteriography, and reduce operation time, radiation dose, and postoperative recurrence rate [8]. Although bronchoscopy and CTA are similarly adequate for the localization of hemoptysis, CTA is more accurate in determining the mechanism and cause of this condition [8]. A diameter of the bronchial artery larger than 2 mm is considered pathological [9, 10]. However, the culprit bronchial artery does not necessarily need to have a wider diameter [11]. Moreover, there is no significant correlation between the diameter of the bronchial artery and the amount of hemoptysis [12]. During CTA imaging, the pressure and amount of contrast agents injected into the arterioles are lower than during digital subtraction angiography (DSA). Therefore, the detection rate of small arterial hemorrhage, pulmonary arterial shunt, ectopic bronchial artery, and lateral branch blood supply is lower in CTA than in DSA [13]. In the presented case, the diameter of the tube of bronchial artery 2 measured by CTA was increased to 2 mm, but no bleeding was found by DSA. Additionally, CTA did not detect an abnormality of bronchial artery 3, but DSA showed that the diameter of the right lower bronchial artery was enlarged, the branch was disordered, and there were parenchymal staining manifestations. DSA is the gold standard for the diagnosis of bronchial artery malformation, and BAE can be performed simultaneously to block the related blood flow and maintain the lung function of the affected side. DSA imaging should take into account the complex sources of blood-supplying arteries involved in systemic circulation-pulmonary circulation shunt and identify the possible origin of the bronchial artery with the highest possible accuracy. However, despite the improvement in the rate of success, the recurrence after BAE surgery remains high, with early recurrence accounting for 10–29% and long-term recurrence for 10–60% [14]. Risk factors for early rebleeding are associated with incomplete embolization, and risk factors for the late recurrence are related to the recanalization of the same vessel and the formation of new collateral circulation [15]. The interventional treatment of massive hemoptysis should not only prevent the failure to detect the emboli but also consider the possibility of some hidden small vessels reexpanding after the redistribution of blood flow.

Fiberoptic bronchoscopy is an important tool for the diagnosis and treatment of hemoptysis. It enables direct visualization of the trachea and the endobronchial lining, identification of the bleeding site, clearing the clot, directly stopping the bleeding locally, keeping the airway open, and creating conditions permitting further examination and treatment [16]. In addition, bronchoscopy can assist in the diagnosis by facilitating cytology, histology, and immunology analyses. Bronchoscopy is indispensable when the positive diagnosis of hemoptysis is difficult, and when the location of bleeding cannot be detected on CTA, particularly in chronic bilateral lung diseases such as bronchiectasis. Finally, bronchoscopy can be used for local control of hemoptysis and airway hygiene before surgery. Limitations inherent in the use of bronchoscopy include the difficulty in defining the specific blood vessels responsible for bleeding and certain risks in the procedure when the bleeding is large and active [16]. Moreover, some cases of hemoptysis do not show active bleeding under fiberoptic bronchoscopy. In the presented case, bronchial artery embolization was performed first, and bronchoscopy was performed after the bleeding was stopped; this approach resulted in a quick recovery of the child.

Massive hemoptysis is a potentially fatal disease, which merits further research aiming at the development of approaches for its timely control. The mortality rate of hemoptysis depends mostly on the underlying etiology and the magnitude of bleeding. Therefore, prompt identification of the etiology and the site of hemoptysis is critical [17]. Although rare, bronchial artery malformation should be considered in children presenting with moderate-to-severe intermittent hemoptysis in the absence of other identifiable causes. In the reported case, the patient also had an ectopic bronchial artery originating from the right renal artery, which is rarely encountered in clinical practice and easy to be missed. Misdiagnosis or delayed diagnosis of pulmonary vascular malformations may result in unnecessary treatment, hospitalization, and even death. When managing massive hemoptysis, it is essential to recognize that time is of the essence, and every second lost increases the number of alveoli flooded with blood.

## Data Availability

The data used to support the findings of this study are available from the corresponding author upon request.

## Abbreviations

CTA: Computed tomography angiography
CT: Computed tomography
BAE: Bronchial artery embolization.
